# Profiling of metastatic small intestine neuroendocrine tumors reveals characteristic miRNAs detectable in plasma

**DOI:** 10.18632/oncotarget.16908

**Published:** 2017-04-07

**Authors:** Michaela Bowden, Chensheng W. Zhou, Sui Zhang, Lauren Brais, Ashley Rossi, Laurent Naudin, Arunthi Thiagalingam, Ewa Sicinska, Matthew H. Kulke

**Affiliations:** ^1^ Department of Medical Oncology, Dana-Farber Cancer Institute, Boston, MA, USA; ^2^ IPSEN Innovation, Les Ulis, France; ^3^ IPSEN Bioscience Inc., Cambridge, MA, USA

**Keywords:** SINET, microRNA, plasma

## Abstract

**Background:**

Current diagnostic and prognostic blood-based biomarkers for neuroendocrine tumors are limited. MiRNAs have tumor-specific expression patterns, are relatively stable, and can be measured in patient blood specimens. We performed a multi-stage study to identify and validate characteristic circulating miRNAs in patients with metastatic small intestine neuroendocrine tumors, and to assess associations between miRNA levels and survival.

**Methods:**

Using a 742-miRNA panel, we identified candidate miRNAs similarly expressed in 19 small intestine neuroendocrine tumors and matched plasma samples. We refined our panel in an independent cohort of plasma samples from 40 patients with metastatic small intestine NET and 40 controls, and then validated this panel in a second, large cohort of 120 patients with metastatic small intestine NET and 120 independent controls.

**Results:**

miRNA profiling of 19 matched small intestine neuroendocrine tumors and matched plasma samples revealed 31 candidate miRNAs similarly expressed in both tissue and plasma. We evaluated expression of these 31 candidate miRNAs in 40 independent cases and 40 normal controls, and identified 4 miRNAs (miR-21-5p, miR-22-3p, miR-29b-3p, and miR-150-5p) that were differently expressed in cases and controls (p<0.05). We validated these 4 miRNAs in a separate, larger panel of 120 cases and 120 controls. We confirmed that high circulating levels of miR-22-3p (p<0.0001), high levels of miR 21-5p, and low levels of miR-150-5p (p=0.027) were associated with the presence of metastatic small intestine NET. While levels of 29b-3p were lower in cases than in controls in both the initial cohort and the validation cohort, the difference in the validation cohort did not reach statistical significance. We further found that high levels of circulating miR-21-5p, high levels of circulating miR-22-3p and low levels of circulating miR-150-5p were each independently associated with shorter overall survival. A combined analysis using all three markers was highly prognostic for survival (HR 0.47, 95% CI 0.27-0.82).

**Conclusions:**

Our study suggests that elevated circulating levels of miR-21-5p and miR-22-3p and low levels of miR-150-5p are characteristic in patients with metastatic small intestine neuroendocrine tumors, and further suggests that levels of these miRNAs are associated with overall survival. These observations provide the basis for further validation studies, as well as studies to assess the biological function of these miRNAs in small intestine neuroendocrine tumors.

## INTRODUCTION

Accurate, blood-based biomarkers to facilitate diagnosis and to monitor neuroendocrine tumor response to treatment are currently limited. Chromogranin A, a protein secreted by neuroendocrine cells, is a commonly used biomarker in patients with neuroendocrine tumors and can be obtained at diagnosis or in follow up of patients with resected or metastatic disease [1]. However, chromogranin A has limited sensitivity and specificity in this setting [2–5]. Measurement of plasma or urinary 5-HIAA, a serotonin metabolite is also used as a clinical tool in the care of patients with neuroendocrine tumors [6, 7]. While measurement of 5-HIAA is useful in patients whose neuroendocrine tumors secrete serotonin, it has only limited utility in other contexts.

A need for new biomarkers in neuroendocrine tumors was highlighted as a key unmet need at consensus meetings sponsored by the National Cancer Institute in 2007 and 2011 and at a recent multinational expert consensus panel in 2015 [8–10]. MiRNAs are short (approximately 22nt’s) RNA sequences that have been shown to broadly regulate gene expression at a post-transcriptional level, by binding to the 3’ region of target RNAs, resulting in mRNA degradation and inhibition of translation [11]. MiRNAs are relatively stable in human tumor samples and can also be readily measured in blood specimens [12]. Their stability allows for the measurement of miRNAs across large numbers of specimens.

MiRNAs expression patterns furthermore appear to be specific to tumor type. In an initial study evaluating 217 miRNAs in 334 diverse human cancer samples, miRNA expression profiles were highly correlated with specific tumor lineages [13]. Measurement of miRNA expression patterns in blood specimens has also been shown to correlate with clinical outcomes. Studies in lung, NSCLC and ovarian cancer have identified specific serum miRNA signatures that correlate with survival [14–17]. The utility of miRNAs as biomarkers in patients with neuroendocrine tumors, however, remains uncertain [18]. Preliminary studies have suggested that miRNA patterns in neuroendocrine tumor tissue may correlate with tumor grade and stage [19, 20]. A more recent study revealed that, in patients with small intestine neuroendocrine tumors, tissue miRNAs can be detected in serum samples, and that serum levels may correlate with tumor stage and with treatment status [21].

To further define characteristic miRNAs in the serum of patients with neuroendocrine tumors, we undertook a multi-stage study; focusing on neuroendocrine tumors of the small intestine (SINET). We first developed a panel of candidate miRNAs detectable in patient plasma, based on evaluation of SINET samples and matched plasma samples. We refined our panel in an independent cohort of 40 cases and 40 controls and then validated this panel in a second, large cohort of 120 patients and 120 matched independent controls. Finally, we assessed whether the identified miRNAs in our panel were associated with clinical outcomes.

## RESULTS

### Identification of candidate miRNAs

To develop an initial candidate panel of characteristic miRNAs detectable in the plasma of patients with small intestine neuroendocrine tumors, we used a strategy in which we identified miRNAs similarly expressed in both tumor tissue and corresponding plasma samples. To perform this analysis, we identified 19 patients with small intestine neuroendocrine tumors, for whom both tumor tissue and paired plasma samples were available. Our screening cohort comprised 14 metastatic and 5 primary SINET cases (see Supplementary Table 1A).

**Figure 1:**
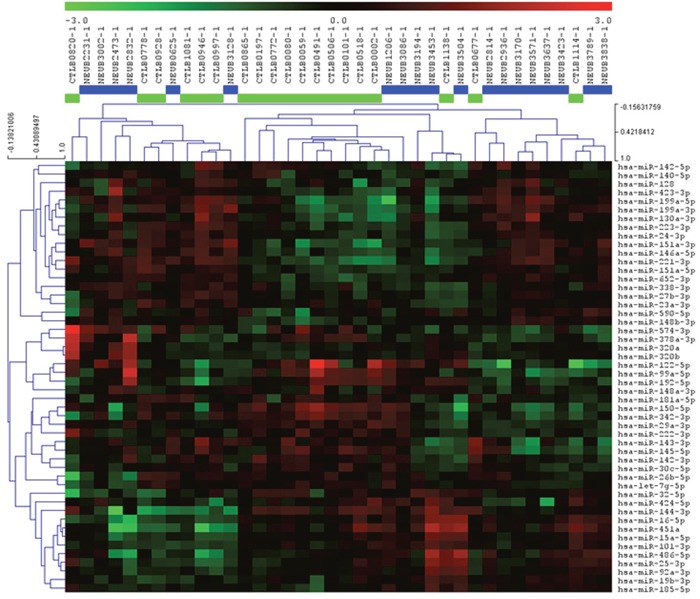
Heatmap and unsupervised hierarchical clustering The heat map shows the result of the two-way hierarchical clustering of miRNAs and samples. Each row represents a single miRNA, and each column represents one sample. The miRNA clustering tree is shown on the left. The color scale shown at the top illustrates the relative expression level of a single miRNA across all samples: red color represents an expression level above mean, green color represents expression lower than the mean. The clustering is performed on all samples, and on the top 50 miRNAs with highest standard deviation. Normalized (dCt) values have been used for the analysis (n=38).

We focused initially on analysis of the plasma samples. We profiled the 19 plasma samples from SINET patients, together with 19 age and gender-matched normal controls using the 742-miRNA panel (Exiqon, Inc). The panel contains many prominent miRNAs; however it is not exhaustive in terms of the total number of detectable microRNAs that have been discovered. We identified 64 miRNAs common to all plasma samples, with an average of 194 miRNAs detectable per sample. Expression levels were normalized to the mean expression level in the combined cohort (n=38). A supervised analysis of the ranked top 50 differentially expressed miRNAs demonstrated potential for clustering between cases and controls, suggesting the presence of characteristic miRNAs either over or under-expressed in SINET patients (Figure 1).

To further refine our list of candidate miRNAs, we next performed miRNA sequencing on the 19 paired tissue specimens. 519 miRNAs were detected in all tissue samples, with an average of 708 miRNAs detectable per sample. We normalized expression levels to the overall mean miRNA expression level in the 19 samples. We then correlated the miRNA expression data from the tumor samples with the miRNA profiles identified in the patient blood samples, with the goal of identifying characteristic miRNAs differentially expressed in a parallel fashion in both tissue and blood.

**Figure 2:**
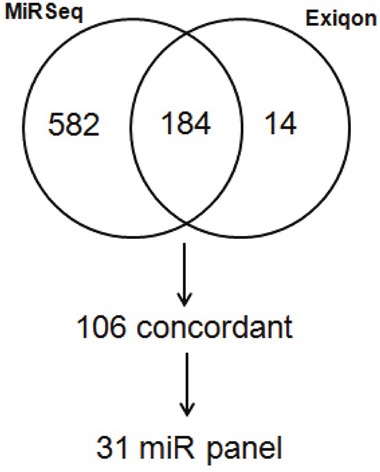
Venn diagram comparison of binned scores from normalized generated from Exiqon profiling of blood and miRNA sequencing of matched tumor tissue from metastatic SINET patients

In identifying these candidate miRNAs, we limited our analysis to 184 miRNAs that were detected in at least 10/19 plasma and matching tumor tissue samples. We then binned normalized expression levels into quartiles for both data sets. Of the 184 miRNAs evaluated, 106 were concordant based on the binned score comparison as shown in Figure 2. In total 31 candidate miRNAs were identified (see Supplementary Table 2).

### Identification of an miRNA panel of characteristic miRNAs detectable in plasma of patients with metastatic small intestine NETs

**Figure 3:**
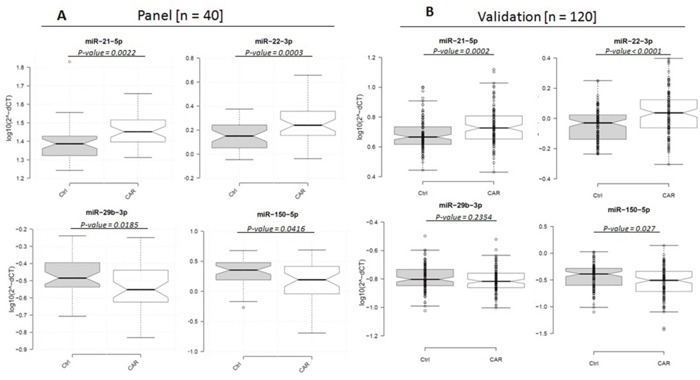
Box plots of relative expression (2-ΔCt) levels of miR-21-5p, miR-22-3p, miR-29b-3p and miR-150-5p in 40 matched metastatic SINET and healthy control plasma samples (log_10_ scale on Y-axis) Center lines show the medians; box limits indicate the 25th and 75th percentiles; whiskers extend 1.5 times the interquartile range from the 25th and 75th percentiles, outliers are represented by dots. n = 40 sample points measured as part of the follow-up panel (right); n = 111 and n=110 sample data points for the patient and controls, respectively as part of the validation cohort (left), where individual data points are included.

To develop the final miRNA panel, we evaluated expression levels of the 31 previously identified candidate miRNAs in plasma samples from an independent group of 40 cases of metastatic SINETs and 40 normal healthy controls (see Supplementary Table 1B). Among the 31 candidate miRNAs, we identified 4 miRNAs; hsa-miR-22-3p, hsa-miR-21-5p, hsa-miR-29b-3p and hsa-miR-150-5p that were differently expressed between the patient and control groups at significance level of < 0.05. Levels of miR-21-5p and miR-22-3p were higher, and miR-29b-3p and miR-150-5p were lower in the plasma of the 40 metastatic small bowel NET patients compared to the 40 matched healthy controls (Figure 3A).

### Validation of the 4-miRNA panel in cases and controls

We then validated miR-21-5p, miR-22-3p, miR-29b-3p and miR-150-5p in the plasma from a second, large independent cohort of 120 metastatic SINET patients and 120 matched normal healthy controls (see Supplementary Table 1C). As part of the experimental design we included 2 general tissue markers (miR-103-3p and miR-191-5p), 2 hemolytic markers (miR-23a-3p and miR-451a) and 2 internal normalization markers (miR-29c-3p and miR-425-5p). 9 SINET patient and 10 control samples were excluded from the analysis based on evidence of hemolysis. We confirmed that expression of 3 of the 4 miRNAs in our panel were significantly different between the patient and control groups. As in the previous cohort, we observed up-regulation of miR-21-5p and miR-22-3p and down-regulation of miR-150-5p in patients with metastatic SINETs (Figure 3B). Both miR-21 and miR-22 reside on chromosome 17 and are positively correlated with each other (r: 0.41, p<.0001, Supplementary Table 3). miR-150 was strongly negatively correlated with miR-22 (r: -0.47, p<0.0001, Supplementary Table 3). The fourth miRNA, miR-29b-3p was downregulated compared to normal controls, though the difference did not reach statistical significance.

### Association of the 4-miRNA panel with NET clinical features

To explore the potential utility of our 4 miRNA panel as a clinical tool, we first assessed the association of expression of the 4 miRNAs with standard clinical parameters in the 111 SINETs patients (Table 1). Expression levels were dichotomized into low and high using the overall patient median as the cutoff. The median expression level was utilized as the cut-off to reduce possible data-driven bias. We found no differences in expression based on age at blood draw, patient gender and systemic treatment prior to blood draw (treatment naive or otherwise). Minor differences in expression levels were observed based on ethnicity (white or otherwise) and tumor differentiation; however results were not conclusive given the small size of the subgroups within the cohort. No significant difference was observed between the miRNA expression levels and whether patients were receiving treatment with a somatostatin analog.

**Table 1:** Clinical characteristics of the metastatic SINET patients dichotomized into low and high expression levels using patient median as the cutoff

Number of patients (%)								
Variables	miR-21		miR-22		miR-29b		miR-150	
	High	Low	High	Low	High	Low	High	Low
Age								
Median	63.2	63.7	64.2	63.2	64.4	63.1	63.4	63.2
Range	31.0-84.3	28.9-80.7	35.4-80.9	28.9-84.3	28.9-84.3	32.5-80.9	28.9-84.3	32.5-80.9
Gender								
Female	30 (53.6)	24 (43.6)	28 (50.0)	26 (47.3)	22 (40.0)	32 (57.1)	32 (57.1)	22 (40.0)
Male	26 (46.4)	31 (56.4)	28 (50.0)	29 (52.7)	33 (60.0)	24 (42.9)	24 (42.9)	33 (60.0)
Race								
White	49 (87.5)	50 (90.9)	51 (91.1)	48 (87.3)	52 (94.5)	47 (83.9)	50 (89.3)	49 (89.1)
Other	7 (12.5)	5 (9.1)	5 (8.9)	7 (12.7)	3 (5.5)	9 (16.1)	6 (10.7)	6 (10.9)
Differentiation								
Well	53 (94.6)	52 (94.5)	53 (94.6)	52 (94.5)	51 (92.7)	54 (96.4)	52 (92.9)	53 (96.4)
Other	3 (5.4)	3 (5.5)	3 (5.4)	3 (5.5)	4 (7.3)	2 (3.6)	4 (7.1)	2 (3.6)
Octreotide								
Yes	34 (60.7)	26 (47.3)	34 (60.7)	26 (47.3)	27 (49.1)	33 (58.9)	28 (50.0)	32 (58.2)
No	22 (39.3)	29 (52.7)	22 (39.3)	29 (52.7)	28 (50.9)	23 (41.1)	28 (50.0)	23 (41.8)

### Association of miRNA expression and prognosis

To further assess the potential clinical relevance of the miRNAs identified in our study, we evaluated whether the level of miRNA expression in plasma was associated with overall survival (OS) in the validation cohort (n = 97 SINET patients, where follow-up was available). 14 SINET patients had no follow-up post-blood draw and were therefore excluded from the subsequent analysis. We assessed survival associations from time of blood draw to time of death or censoring, adjusting for age at blood draw, patient gender, ethnicity (white or otherwise), tumor differentiation (well or otherwise), and any met treatment prior to blood draw (treatment naive or otherwise). The median follow-up time was 4.3 years.

**Figure 4:**
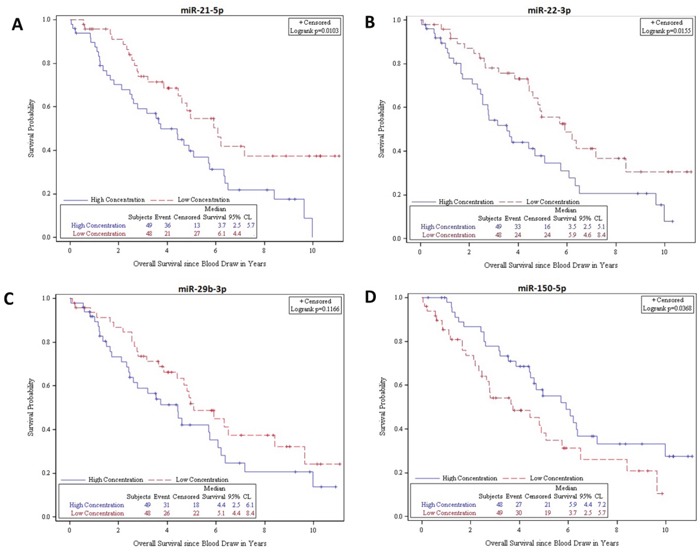
Kaplan-Meier survival product limit estimates for 97 SINET patients with metastatic disease in the validation cohort for plasma miR-21-5p, miR-22-3p, miR-29b-3p and miR-150-5p levels The p-value was calculated using the log-rank test between patients with high and low expression.

**Figure 5:**
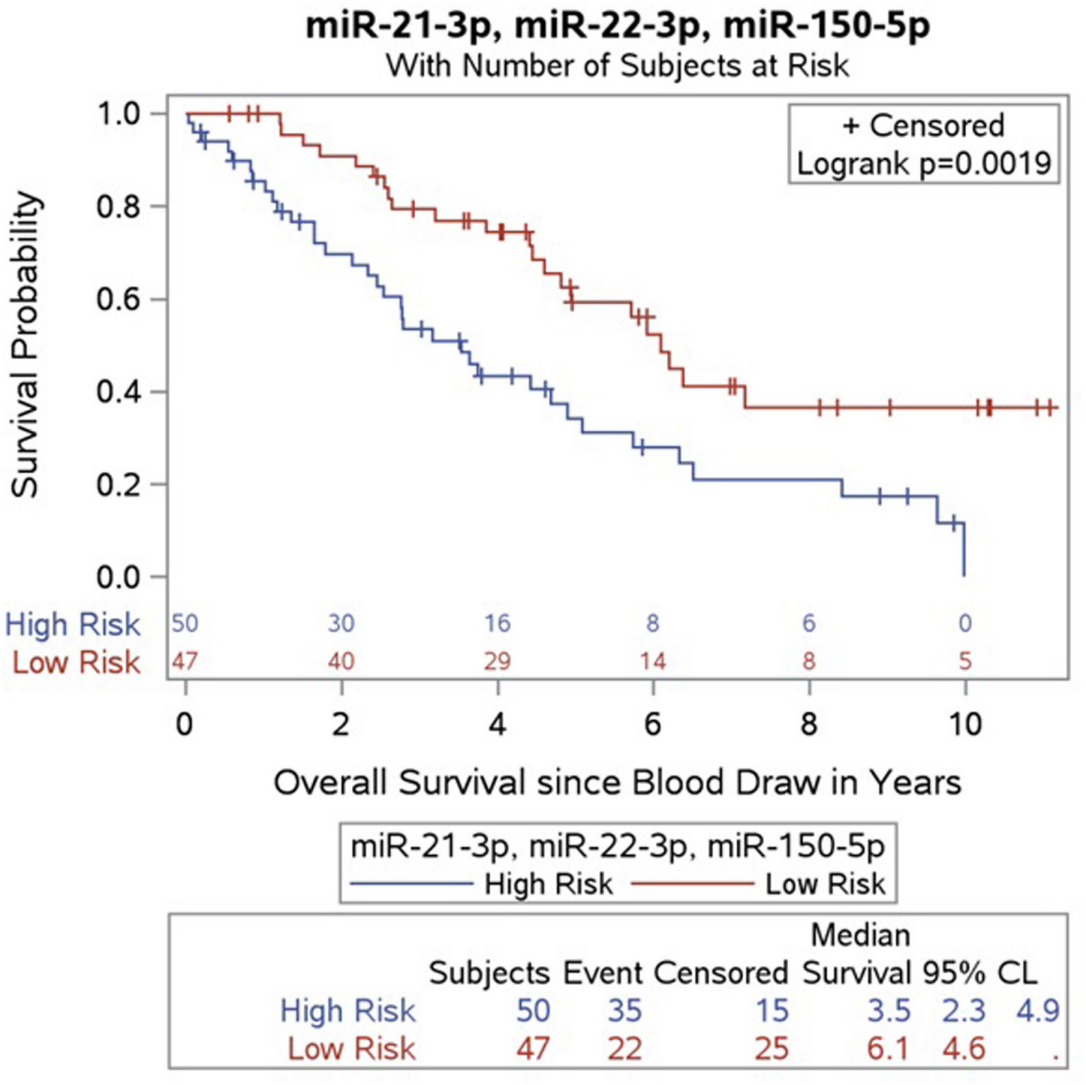
Kaplan-Meier survival product limit estimates for 97 SINET patients with metastatic disease in the validation cohort for plasma combining miR-21-5p, miR-22-3p levels and miR-150-5p levels The p-value was calculated using the log-rank test between patients with high and low expression. The high risk population includes patients with at least 2 positive out of the 3 biomarkers.

Using Kaplan-Meier and log rank methods, we found that low plasma expression of miR-21-5p (P=0.0103) and miR-22-3p (P=0.0155) and high expression of miR-150-5p (P=0.0368) were significantly associated with prolonged OS (Figure 4). We found that elevated plasma expression of miR-29b-3p was associated with a trend towards improved survival, though this did not reach statistical significance (P=0.1166). Multivariate analysis, as shown in Table 2, confirmed that high expression of plasma miR-21-5p and miR-22-3p was independently associated with shorter survival (miR-21-5p - HR: 0.54; 95% CI, 0.31-0.96; P=0.0345; miR-22-3p - HR; 0.56; 95% CI, 0.32-0.96; P=0.0362) whereas high expression of plasma miR-150-5p was associated with longer survival (HR: 2.24; 95% CI; 1.18-4.25; P=0.0136). Additionally we generated a new high/low risk index to combine miR-21-5p, miR-22-3p and miR-150-5p, considering high concentration for miR-21-3p and miR-22-3p and low concentration for miR-150-5p as high risk to further analyze their prognostic value as a 3-miR signature. The new index strengthened both the univariate (P=0.0025) and multivariate (P=0.0084) analysis. The combination of the 3 miRNA's was associated with a larger HR associated with shorter survival (HR: 0.47; 95% CI, 0.27-0.82) as shown in Figure 5. Elevated plasma CgA levels have been associated with poor overall prognosis in patients with advanced disease (Ter-Minassian, et al 2014). We evaluated the prognostic value in a model that combined CgA with the 3 miRNA's signature, and observed an even stronger HR of 0.41 (95% CI, 0.2-0.85), with a significant association with shorter overall survival (P=0.0171). CgA concentration was dichotimized using 2x the upper limit of the range, (0.6-39 ug/L) as a cutoff for elevated expression.

**Table 2:** Univariate and multivariate analyses of plasma miR-21-5p, miR-22-3p, miR-29b-3p and miR-150-5p association with overall survival in metastatic SINET patients

MiRNA	Expression	# Death/at risk	Univariate analysis			Multivariate analysis			# Death/at risk	Multivariate analysis + CgA		
			HR	95% CI	P-value	HR	95% CI	P-value		HR	95% CI	P-value
miR-21-5p	High	36/49	ref			ref			21/31	ref		
	Low	21/48	0.5	0.29-0.86	0.0118	0.54	0.31-0.96	0.0345	13/39	0.62	0.29-1.32	0.2128
miR-22-3p	High	33/49	ref			ref			21/35	ref		
	Low	24/48	0.53	0.31-0.89	0.0173	0.56	0.32-0.96	0.0362	13/35	0.45	0.22-0.93	0.0315
miR-150-5p	High	27/48	ref			ref			17/36	ref		
	Low	30/49	1.75	1.03-2.97	0.0392	2.24	1.18-4.25	0.0136	17/34	4.4	1.69-11.46	0.0024
miR-29b-3p	High	31/49	ref			ref			18/36	ref		
	Low	26/48	0.66	0.39-1.11	0.1192	0.63	0.36-1.08	0.0951	16/34	0.62	0.29-1.32	0.2135
3-miR*	High	35/50	ref			ref			21/33	ref		
	Low	22/47	0.44	0.25-0.75	0.0025	0.47	0.27-0.82	0.0084	13/37	0.41	0.2-0.85	0.0171

*Combination includes **high** miR-21-5p, **high** miR-22-3p and **low** miR150-5p but not miR29b-3p.

We additionally assessed the 3 microRNA's potential diagnostic utility. The highest AUC value from ROC estimates was calculated as 0.7025, when miR-21-5p, miR-22-3p and miR-150-5p were combined, with an ROC of 0.5 indicating no ability to discriminate between cases and controls, and an ROC of 1 indicating perfect discrimination between cases and controls.

## DISCUSSION

In this study, we used a multi-step process to identify and validate characteristic miRNAs either over or under-expressed in the plasma of patients with metastatic small intestine NETs. Following the identification of 31 candidate miRNAs using paired plasma and tumor specimens, we identified 4 characteristically over or under-expressed miRNAs by comparing miRNA profiles in plasma from cases and normal controls. We then validated this panel in a separate, independent cohort of 111 cases and 110 controls. We have shown that elevated levels of miR-21-5p and miR-22-3p and low levels of miR-150-5p are found in metastatic small bowel neuroendocrine disease. We further found that expression of these miRNAs had prognostic significance: high plasma expression of miR-21-5p and miR-22-3p; and low expression of miR-150-5p were associated with poor overall survival.

The identification of novel biomarkers in neuroendocrine tumors has proved challenging. Chromogranin A, a protein secreted from secretory granules in neuroendocrine cells, has become a standard biomarker in patients with neuroendocrine tumors despite having only limited utility. While chromogranin A has, in some studies, been associated with clinical outcomes, chromogranin A assays are highly variable [22, 23]. (Jensen, et al. 2013). Furthermore, levels of circulating Chromogranin A can be elevated in the setting of proton pump inhibitor use and in a number of non-malignant conditions including chronic renal failure and hepatic disease [3, 24]. Measurement of the serotonin metabolite 5-HIAA in blood or urine also has relatively limited utility, as only a minority of neuroendocrine tumors are associated with serotonin secretion.

Recent interest has focused on the use of blood-based markers based on neuroendocrine tumor genomics. A potential challenge with this approach is the relative genomic stability of neuroendocrine tumors, which precludes biomarkers based on the identification of characteristic genetic mutations. Whole genome profiling of small intestine neuroendocrine tumors revealed recurrent mutations in the cyclin dependent kinase inhibitor CDKN1B in only 8% of cases [25]. Pancreatic neuroendocrine tumors are also characterized by recurrent mutations in a relatively limited number of genes, which include tumor suppressor gene *MEN1*, as well as *ATRX* and *DAXX*, genes implicated in chromatin remodeling [26]. Preliminary studies have suggested that a multianalyte approach, using a 51-transcript mRNA panel, may be more sensitive and specific than currently available biomarkers [27–29] though this assay has yet to be validated in large, prospective studies.

The measurement of circulating miRNAs represents a potentially promising approach for the development of more sensitive and specific biomarkers in neuroendocrine tumors. Initial tissue-based studies have suggested that neuroendocrine tumors have characteristic miRNA expression profiles. An initial analysis of 8 primary and 6 metastatic neuroendocrine tumors, using a 95-miRNA panel, suggested down-regulation of miR-133a in metastatic lesions [20] (Ruebel et al. 2010). Our miR-Seq results corroborate previous SINET-related miR tissue-based studies, where miR-133a has frequently been downregulated in the metastatic tumors in comparison to the primary tumors [19, 21, 30]. Miller also reported miR-1 and miR-143-3p were also downregulated and found NUAK2 and FOSB target genes were functionally crucial in the progression of SINETs. We too saw downregulation of miR-1 and miR-143-3p in our study in the metastatic tumors compared to the primary SINET tumors (data not shown).

Studies of circulating miRNAs in plasma however remain limited. Our observations that elevated levels of miR-21-5p and miR-22-3p and low levels of miR-150-5p are found in metastatic small bowel neuroendocrine disease but also have prognostic value support the further investigation of circulating miRNAs in neuroendocrine tumor disease. The observation that miR-21 is highly expressed in the plasma of patients with metastatic neuroendocrine tumors is consistent with its known function and with findings in other malignancies [2]. Expression of miR-21 has been shown to be associated with proliferation, apoptosis and migration [31] and involved in invasion, intravasation and metastasis [32, 33]. miR-21 is upregulated in glioblastoma [34], breast cancer [35, 36], lung cancer [37], gastric cancer [38], colorectal cancer [39], hepatocellular carcinoma [40], pancreatic cancer [41] as well as other tumor types [2]. Our finding that high expression of miR-21 is associated with shorter overall survival in metastatic small bowel NET patients, is also consistent with recent reports in metastatic breast [36], gastric, and cervical cancer [42, 43].

From the approximate 100 target genes that have been validated experimentally to be regulated by miR-21-5p (Supplementary Table 4), a number of key genes are known to be important in SINET tumorigenesis, including PDCD4, PTEN, AKT2, BCL-2 and SERPINB5. PTEN has an important role in NET biology, regulating the activity of MTOR through the AKT pathway, where its downregulated expression has been associated with shorter disease-free and overall survival [44, 45]. Suppressed PTEN has further been linked to miR-21 overexpression in pulmonary and pancreatic NETs [46–48]. Furthermore Chan et al reviewed the importance of the MTOR pathway in NET pathogenesis and reported on the testing of rapamycin and everolimus in Phase II trials [49]. PDCD4, located at 11q13 close to MEN1, has been found to be absent in NETs and has been shown to act as a tumor suppressor in neuroendocrine cells [50]. BCL-2, regulated by miR-21, was found to be predominantly negatively expressed in SINET tissues by IHC. Methylation of SERPINB5, another target gene of miR-21, is associated with reduced mRNA expression levels in relation to the loss of Chr18 in SINETs [51, 52]. We further found elevated expression of circulating miR-22 was associated both with the presence of metastatic small intestine NET and with shorter overall survival in this setting. The role of miR-22 in cancer development remains uncertain [53]. miR-22 is highly conserved across species, and has been shown to regulate phosphatase and tensin homologue tumor suppressor gene, PTEN [54]. MiR-22 was originally described as having a tumor suppressor function in several human malignancies, including breast [55], liver [56], colon [57] and ovarian cancer [58]. Other studies, however, have suggested it may also have an oncogenic function. Circulating miR-22 was found to be upregulated in NSCLC patients where it was also associated with disease progression [59]. miR-22 has also been shown to be upregulated and predictive in meningiomas and in prostate cancer [60, 61]. Our observations suggest that, at least in neuroendocrine tumors, miR-22 may play an oncogenic role. As with miR-21, high expression of miR-22 was associated with shorter overall survival in metastatic small bowel NET patients.

In contrast to our findings with miR-21 and miR-22, we found that low (rather than high) levels of miR-150 were characteristic of metastatic SINET and were associated with shorter overall survival in this setting. These findings are consistent with studies in other malignancies, where miR-150 has predominantly been described as having a tumor suppressor function. In patients with hepatitis B virus, low expression of miR-150 has been associated with a higher risk of HCC development, as well as in shorter survival in patients who develop HCC [62, 63]. In AML, lower levels of miR-150 are associated with disease recurrence [64].

While ROC curves suggested that the 3-miRNA signature was likely to be of only limited value in distinguishing neuroendocrine tumor cases from normal controls, we did find that a model incorporating all three miRNAs had strong prognostic significance. The model was even stronger when CgA was taken into account, suggesting that these miRNA signatures have the potential to be used clinically as prognostic indicators. Given the biological role of miRNAs as regulatory molecules, future studies examining whether these signatures may also have predictive value with regard to response to specific systemic treatments are also warranted.

There are some limitations to our study. Firstly we validated our panel in a large cohort of cases and controls from a single institution; unencumbered by variation in sample handling and processing. Further validation in multi-institutional cohorts with standardized protocols and inclusion of blood draws over the time course of a therapeutic treatment regimen or in the setting of disease progression would be warranted. Secondly there is no single circulating miRNA/small RNA that on its own can be utilized as the “housekeeping” reference across all tumor types. Rather selection of an appropriate normalizer needs to be undertaken for any given studied population [65]. We tested a panel of 5 miRNAs as candidate references, however were dissatisfied with the outcome individually or in combination. We therefore elected to use a global mean of all miRNAs being measured. NormFinder confirmed that the global mean was the most stable candidate reference within our cohorts. Thirdly there is a lack of a real “normal” comparator tissue applicable for SINETs, which makes comparing tissue and plasma profiles challenging. Ultimately, functional studies examining the role of these miRNAs are warranted; however, limited *in vitro* models of NET make such functional studies challenging.

In summary we identified miR-21-5p, miR-22-3p and miR-150-5p as circulating biomarkers associated with metastatic small bowel neuroendocrine tumors. We further found that expression levels of these markers are associated with survival, and this association was strongest when we created a model incorporating the 3-miRNA signature together with CgA. Further studies to evaluate whether these markers can predict response to treatment are warranted. Additionally, functional studies to identify the role of these miRNAs in neuroendocrine tumorigenesis may shed light on the molecular mechanisms driving tumor growth.

## MATERIALS AND METHODS

### Patient and control samples

Neuroendocrine tumor tissue and plasma samples were obtained from the Dana-Farber Cancer Institute Neuroendocrine Tumor Biospecimen database, under an IRB-approved protocol in which patients provide informed consent for collection of tissue specimens obtained during routine clinical care, as well as collection of additional blood specimens for research use. Plasma was also collected from consenting spousal/friend as healthy controls. Briefly blood (up to 10ml) was collected into tubes containing EDTA, and the separation procedure was carried out within 3hr of venipuncture. The samples were spun at 3000rpm for 15min resulting in approximately 3-6ml plasma. The plasma was aliquoted and stored at -80°C for preservation. A portion of each tissue sample was preserved in OCT and evaluated to confirm histological features and tumor cellularity. Additional portions of each tissue were snap frozen and banked for subsequent molecular characterization.

### miRNA profiling in plasma samples

miRNA profiling in plasma samples was performed using the miRCURY LNA (Locked Nucleic Acid) Universal miRNA PCR assays [Exiqon, Vedbaek, Denmark], where all miRNAs are polyadenylated and reverse transcribed into cDNA in a single step and amplification is performed on a Roche Lightcycler 480. A total of 742 miRNAs were assayed. For the RNA extraction of biofluids, 3 RNA spike-ins (UniSp2, UniSp4 and UniSp5) pre-mixed, each at different concentration in 100 fold increments were added. This set of spike-ins was intended as an RNA isolation control (only applicable if Exiqon has performed sample prep). For the reverse transcription step, one spike-in (UniSp6) was added. This control was used to confirm that the reverse transcription and amplification occurs with equal efficiency in all samples. Results were reported as a Ct, which is calculated, as the maximum second derivative of the amplification curve. For generating ΔCt values, we utilized a global mean Ct value of all miRNAs measured as the reference for normalization of the target Ct values generated for each of the miRNAs as measured in the original screen (742 miRNA), the follow-up target panel (31 miRNAs) and the validation set. NormFinder was utilized to identify the optimal normalization miRNA(s) among a set of candidate references [65]. No template controls (NTC’s) were included as negative controls. All Ct values within 5 Ct values of NTC's were automatically omitted from all analysis. The 2^-ΔΔCt^ method was used to analyze the relative expression of miRNAs, and the student t-test to compare the expression between the patient and control groups for the original screen and the follow-up target panel analysis. To monitor hemolysis two miRNAs were used; one that is expressed in red blood cells (miRNA-451) and one that is relatively stable in serum and plasma and is not affected by hemolysis (miRNA-23a).

### miRNA sequencing in tumor samples

Total RNA, enriched for miRNA extractions were performed on snap frozen tissue specimens. H&Es were reviewed to identify enriched regions of high tumor cellularity, although tumors were not microdissected to select only for tumor cells exclusively. Briefly snap frozen tumor was cut and processed using a QIAShredder [Qiagen #79654], and miRNA-enriched total RNA was extracted using MiRNeasy Mini Kit [Qiagen # 217004]. 1μg of RNA was used as input in the ScriptMiner™ Small RNA-Seq Library Preparation Kit [EpiCenter #SMMP101212]. Resulting cDNA libraries had a maximum peak of approximately 140bp. Libraries were QC'd with a Agilent High Sensitivity DNA Kit on a 2100 bioanalyzer system [Agilent #5067-4626]. qPCR library quantification was performed using KAPA SYBR® FAST qPCR Kits [KapaBiosystems, Wilmington, MA]. A final concentration of 10pM was loaded onto v3 Illumina flow cells. Libraries were sequenced as 50bp single end reads on a HiSeq 2200 system. Sequenced reads were aligned to the reference human genome (hg19) using the universal RNA-Sq aligner, STAR algorithm to produce BAM files. MiRNAs were quantified in the sample using the mirDeep2 algorithm, which produces count values for all the known miRNA.

### Statistical analysis for validation

Non-parametric Wilcoxon rank sum test was performed to compare the patient and control groups. Descriptive statistics of patient clinical features were tabulated by the dichotomized expression level of 4-miRNA panel at median, and compared by Wilcoxon rank sum test, chi-square test, or fisher's exact test when applicable. The distribution of survival times by dichotomized 4-miRNA panel were assessed using Kaplan-Meier methods and log-rank tests, and then the association was evaluated by Cox proportional hazards regression. Multivariate models were adjusted for age and blood draw, gender, race (white or otherwise), tumor differentiation, and treatment naive or otherwise prior to blood draw. Proportional hazard assumptions were verified by testing for a nonzero slope of scaled Schoenfeld residuals on ranked failure times. In addition, Pearson's correlation was applied to determine the association between all pairs in the 4-miRNA panel. Analyses were conducted using SAS version 9.4 (SAS Institute, Cary, NC). Two-sided p-values <0.05 were considered statistically significant.

### MiRNA target database

The miRTarBase (Release 6.0 September 2015) was utilized to assess the miRNA-21-5p - target interactions (MTIs). 99 genes were found to have been validated experimentally by reporter assay, western blot and microarray, which together are characterized as strong evidence of an interaction [67].

## SUPPLEMENTARY MATERIALS FIGURES AND TABLES







## Abbreviations

5-HIAA: 5-Hydroxyindoleacetic acid
SINET: small intestine neuroendocrine tumor
EDTA: Ethylenediaminetetraacetic acid
OCT: optimal cutting temperature compound
LNA: locked nucleic acid
PCR: polymerase chain reaction
BAM: binary alignment/map
OS: overall survival
HR: hazard ratio
AUC: area under the curve
ROC: receiver operating characteristic
NSCLC: non-small cell lung carcinoma
AML: acute myeloid leukemia
HCC: hepatocellular carcinoma
CgA: chromogranin A

## References

[1] Kulke MH, Shah MH, Benson AB, Bergsland E, Berlin JD, Blaszkowsky LS, Emerson L, Engstrom PF, Fanta P, Giordano T, Goldner WS, Halfdanarson TR, Heslin MJ (2015). Neuroendocrine tumors, version 1.2015. J Natl Compr Canc Netw.

[2] Allegra A, Alonci A, Campo S, Penna G, Petrungaro A, Gerace D, Musolino C (2012). Circulating microRNAs: new biomarkers in diagnosis, prognosis and treatment of cancer (review). Int J Oncol.

[3] Marotta V, Nuzzo V, Ferrara T, Zuccoli A, Masone M, Nocerino L, Del Prete M, Marciello F, Ramundo V, Lombardi G, Vitale M, Colao A, Faggiano A (2012). Limitations of Chromogranin A in clinical practice. Biomarkers.

[4] Modlin IM, Gustafsson BI, Moss SF, Pavel M, Tsolakis AV, Kidd M (2010). Chromogranin A--biological function and clinical utility in neuro endocrine tumor disease. Ann Surg Oncol.

[5] Yao JC, Pavel M, Phan AT, Kulke MH, Hoosen S, St Peter J, Cherfi A, Oberg KE (2011). Chromogranin A and neuron-specific enolase as prognostic markers in patients with advanced pNET treated with everolimus. J Clin Endocrinol Metab.

[6] Bhattacharyya S, Jagroop A, Gujral DM, Hayward C, Toumpanakis C, Caplin M, Mikhailidis DP, Davar J (2013). Circulating plasma and platelet 5-hydroxytryptamine in carcinoid heart disease: a pilot study. J Heart Valve Dis.

[7] Dobson R, Burgess MI, Banks M, Pritchard DM, Vora J, Valle JW, Wong C, Chadwick C, George K, Keevil B, Adaway J, Ardill J, Anthoney A (2013). The association of a panel of biomarkers with the presence and severity of carcinoid heart disease: a cross-sectional study. PLoS One.

[8] Kulke MH, Siu LL, Tepper JE, Fisher G, Jaffe D, Haller DG, Ellis LM, Benedetti JK, Bergsland EK, Hobday TJ, Van Cutsem E, Pingpank J, Oberg K (2011). Future directions in the treatment of neuroendocrine tumors: consensus report of the National Cancer Institute neuroendocrine tumor clinical trials planning meeting. J Clin Oncol.

[9] Modlin IM, Gustafsson BI, Moss SF, Pavel M, Tsolakis AV, Kidd M (2010). Chromogranin A—biological function and clinical utility in neuro endocrine tumor disease. Ann Surg Oncol.

[10] Oberg K, Modlin IM, De Herder W, Pavel M, Klimstra D, Frilling A, Metz DC, Heaney A, Kwekkeboom D, Strosberg J, Meyer T, Moss SF, Washington K (2015). Consensus on biomarkers for neuroendocrine tumour disease. Lancet Oncol.

[11] Iorio MV, Croce CM (2009). MicroRNAs in cancer: small molecules with a huge impact. J Clin Oncol.

[12] Shen J, Stass SA, Jiang F (2013). MicroRNAs as potential biomarkers in human solid tumors. Cancer Lett.

[13] Lu J, Getz G, Miska EA, Alvarez-Saavedra E, Lamb J, Peck D, Sweet-Cordero A, Ebert BL, Mak RH, Ferrando AA, Downing JR, Jacks T, Horvitz HR, Golub TR (2005). MicroRNA expression profiles classify human cancers. Nature.

[14] Chen X, Hu Z, Wang W, Ba Y, Ma L, Zhang C, Wang C, Ren Z, Zhao Y, Wu S, Zhuang R, Zhang Y, Hu H (2012). Identification of ten serum microRNAs from a genome-wide serum microRNA expression profile as novel noninvasive biomarkers for nonsmall cell lung cancer diagnosis. Int J Cancer.

[15] Hausler SF, Keller A, Chandran PA, Ziegler K, Zipp K, Heuer S, Krockenberger M, Engel JB, Honig A, Scheffler M, Dietl J, Wischhusen J (2010). Whole blood-derived miRNA profiles as potential new tools for ovarian cancer screening. Br J Cancer.

[16] Hu Z, Chen X, Zhao Y, Tian T, Jin G, Shu Y, Chen Y, Xu L, Zen K, Zhang C, Shen H (2010). Serum microRNA signatures identified in a genome-wide serum microRNA expression profiling predict survival of non-small-cell lung cancer. J Clin Oncol.

[17] Resnick KE, Alder H, Hagan JP, Richardson DL, Croce CM, Cohn DE (2009). The detection of differentially expressed microRNAs from the serum of ovarian cancer patients using a novel real-time PCR platform. Gynecol Oncol.

[18] Kentwell J, Gundara JS, Sidhu SB (2014). Noncoding RNAs in endocrine malignancy. Oncologist.

[19] Li SC, Essaghir A, Martijn C, Lloyd RV, Demoulin JB, Oberg K, Giandomenico V (2013). Global microRNA profiling of well-differentiated small intestinal neuroendocrine tumors. Mod Pathol.

[20] Ruebel K, Leontovich AA, Stilling GA, Zhang S, Righi A, Jin L, Lloyd RV (2010). MicroRNA expression in ileal carcinoid tumors: downregulation of microRNA-133a with tumor progression. Mod Pathol.

[21] Li SC, Khan M, Caplin M, Meyer T, Oberg K, Giandomenico V (2015). Somatostatin analogs treated small intestinal neuroendocrine tumor patients circulating microRNAs. PLoS One.

[22] de Laat JM, Pieterman CR, Weijmans M, Hermus AR, Dekkers OM, de Herder WW, van der Horst-Schrivers AN, Drent ML, Bisschop PH, Havekes B, Vriens MR, Valk GD (2013). Low accuracy of tumor markers for diagnosing pancreatic neuroendocrine tumors in multiple endocrine neoplasia type 1 patients. J Clin Endocrinol Metab.

[23] Jensen KH, Hilsted L, Jensen C, Mynster T, Rehfeld JF, Knigge U (2013). Chromogranin A is a sensitive marker of progression or regression in ileo-cecal neuroendocrine tumors. Scand J Gastroenterol.

[24] Raines D, Chester M, Diebold AE, Mamikunian P, Anthony CT, Mamikunian G, Woltering EA (2012). A prospective evaluation of the effect of chronic proton pump inhibitor use on plasma biomarker levels in humans. Pancreas.

[25] Francis JM, Kiezun A, Ramos AH, Serra S, Pedamallu CS, Qian ZR, Banck MS, Kanwar R, Kulkarni AA, Karpathakis A, Manzo V, Contractor T, Philips J (2013). Somatic mutation of CDKN1B in small intestine neuroendocrine tumors. Nat Genet.

[26] Jiao Y, Shi C, Edil BH, de Wilde RF, Klimstra DS, Maitra A, Schulick RD, Tang LH, Wolfgang CL, Choti MA, Velculescu VE, Diaz LA, Vogelstein B (2011). DAXX/ATRX, MEN1, and mTOR pathway genes are frequently altered in pancreatic neuroendocrine tumors. Science.

[27] Modlin IM, Drozdov I, Alaimo D, Callahan S, Teixiera N, Bodei L, Kidd M (2014). A multianalyte PCR blood test outperforms single analyte ELISAs (chromogranin A, pancreastatin, neurokinin A) for neuroendocrine tumor detection. Endocr Relat Cancer.

[28] Modlin IM, Drozdov I, Kidd M (2013). The identification of gut neuroendocrine tumor disease by multiple synchronous transcript analysis in blood. PLoS One.

[29] Modlin IM, Aslanian H, Bodei L, Drozdov I, Kidd M (2014). A PCR blood test outperforms chromogranin A in carcinoid detection and is unaffected by proton pump inhibitors. Endocr Connect.

[30] Miller HC, Frampton AE, Malczewska A, Ottaviani S, Stronach EA, Flora R, Kaemmerer D, Schwach G, Pfragner R, Faiz O, Kos-Kudla B, Hanna GB, Stebbing J (2016). MicroRNAs associated with small bowel neuroendocrine tumors and their metastasis. Endocr Relat Cancer.

[31] Frankel LB, Christoffersen NR, Jacobsen A, Lindow M, Krogh A, Lund AH (2008). Programmed cell death 4 (PDCD4) is an important functional target of the microRNA miR-21 in breast cancer cells. J Biol Chem.

[32] Yang CH, Yue J, Pfeffer SR, Handorf CR, Pfeffer LM (2011). MicroRNA miR-21 regulates the metastatic behavior of B16 melanoma cells. J Biol Chem.

[33] Yin C, Zhou X, Dang Y, Yan J, Zhang G (2015). Potential role of circulating MiR-21 in the diagnosis and prognosis of digestive system cancer: a systematic review and meta-analysis. Medicine (Baltimore).

[34] Chan JA, Krichevsky AM, Kosik KS (2005). MicroRNA-21 is an antiapoptotic factor in human glioblastoma cells. Cancer Res.

[35] Iorio MV, Ferracin M, Liu CG, Veronese A, Spizzo R, Sabbioni S, Magri E, Pedriali M, Fabbri M, Campiglio M, Ménard S, Palazzo JP, Rosenberg A (2005). MicroRNA gene expression deregulation in human breast cancer. Cancer Res.

[36] Yang X, Wang X, Shen H, Deng R, Xue K (2015). Combination of miR-21 with circulating tumor cells markers improve diagnostic specificity of metastatic breast cancer. Cell Biochem Biophys.

[37] Markou A, Tsaroucha EG, Kaklamanis L, Fotinou M, Georgoulias V, Lianidou ES (2008). Prognostic value of mature microRNA-21 and microRNA-205 overexpression in non-small cell lung cancer by quantitative real-time RT-PCR. Clin Chem.

[38] Wu J, Li G, Wang Z, Yao Y, Chen R, Pu X, Wang J (2015). Circulating microRNA-21 is a potential diagnostic biomarker in gastric cancer. Dis Markers.

[39] Basati G, Emami Razavi A, Abdi S, Mirzaei A (2014). Elevated level of microRNA-21 in the serum of patients with colorectal cancer. Med Oncol.

[40] Amr KS, Ezzat WM, Elhosary YA, Hegazy AE, Fahim HH, Kamel RR (2016). The potential role of miRNAs 21 and 199-a in early diagnosis of hepatocellular carcinoma. Gene.

[41] Khan K, Cunningham D, Peckitt C, Barton S, Tait D, Hawkins M, Watkins D, Starling N, Rao S, Begum R, Thomas J, Oates J, Guzzardo V (2016). miR-21 expression and clinical outcome in locally advanced pancreatic cancer: exploratory analysis of the pancreatic cancer Erbitux, radiotherapy and UFT (PERU) trial. Oncotarget.

[42] Kim SY, Jeon TY, Choi CI, Kim DH, Kim GH, Ryu DY, Lee BE, Kim HH (2013). Validation of circulating miRNA biomarkers for predicting lymph node metastasis in gastric cancer. J Mol Diagn.

[43] Zhang L, Zhan X, Yan D, Wang Z (2016). Circulating microRNA-21 is involved in lymph node metastasis in cervical cancer by targeting RASA1. Int J Gynecol Cancer.

[44] Oberg K, Casanovas O, Castaño JP, Chung D, Delle Fave G, Denèfle P, Harris P, Khan MS, Kulke MH, Scarpa A, Tang LH, Wiedenmann B (2013). Molecular pathogenesis of neuroendocrine tumors: implications for current and future therapeutic approaches. Clin Cancer Res.

[45] Missiaglia E, Dalai I, Barbi S, Beghelli S, Falconi M, della Peruta M, Piemonti L, Capurso G, Di Florio A, delle Fave G, Pederzoli P, Croce CM, Scarpa A (2010). Pancreatic endocrine tumors: expression profiling evidences a role for AKT-mTOR pathway. J Clin Oncol.

[46] Lee HW, Lee EH, Ha SY, Lee CH, Chang HK, Chang S, Kwon KY, Hwang IS, Roh MS, Seo JW (2012). Altered expression of microRNA miR-21, miR-155, and let-7a and their roles in pulmonary neuroendocrine tumors. Pathol Int.

[47] Roldo C, Missiaglia E, Hagan JP, Falconi M, Capelli P, Bersani S, Calin GA, Volinia S, Liu CG, Scarpa A, Croce CM (2006). MicroRNA expression abnormalities in pancreatic endocrine and acinar tumors are associated with distinctive pathologic features and clinical behavior. J Clin Oncol.

[48] Vicentini C, Fassan M, D’Angelo E, Corbo V, Silvestris N, Nuovo GJ, Scarpa A (2014). Clinical application of microRNA testing in neuroendocrine tumors of the gastrointestinal tract. Molecules.

[49] Chan J, Kulke M (2014). Targeting the mTOR signaling pathway in neuroendocrine tumors. Curr Treat Options Oncol.

[50] Goke R, Gregel C, Goke A, Arnold R, Schmidt H, Lankat-Buttgereit B (2004). Programmed cell death protein 4 (PDCD4) acts as a tumor suppressor in neuroendocrine tumor cells. Ann N Y Acad Sci.

[51] Verdugo AD, Crona J, Starker L, Stålberg P, Åkerström G, Westin G, Hellman P, Björklund P (2014). Global DNA methylation patterns through an array-based approach in small intestinal neuroendocrine tumors. Endocr Relat Cancer.

[52] Nieser M, Henopp T, Brix J, Stoß L, Sitek B, Naboulsi W, Anlauf M, Schlitter AM, Klöppel G, Gress T, Moll R, Bartsch DK, Heverhagen AE (2017). Loss of chromosome 18 in neuroendocrine tumors of the small intestine: the enigma remains. Neuroendocrinology.

[53] Koufaris C, Valbuena GN, Pomyen Y, Tredwell GD, Nevedomskaya E, Lau CH, Yang T, Benito A, Ellis JK, Keun HC (2016). Systematic integration of molecular profiles identifies miR-22 as a regulator of lipid and folate metabolism in breast cancer cells. Oncogene.

[54] Xu XD, Song XW, Li Q, Wang GK, Jing Q, Qin YW (2012). Attenuation of microRNA-22 derepressed PTEN to effectively protect rat cardiomyocytes from hypertrophy. J Cell Physiol.

[55] Patel JB, Appaiah HN, Burnett RM, Bhat-Nakshatri P, Wang G, Mehta R, Badve S, Thomson MJ, Hammond S, Steeg P, Liu Y, Nakshatri H (2011). Control of EVI-1 oncogene expression in metastatic breast cancer cells through microRNA miR-22. Oncogene.

[56] Zhang J, Yang Y, Yang T, Liu Y, Li A, Fu S, Wu M, Pan Z, Zhou W (2010). microRNA-22, downregulated in hepatocellular carcinoma and correlated with prognosis, suppresses cell proliferation and tumourigenicity. Br J Cancer.

[57] Alvarez-Diaz S, Valle N, Ferrer-Mayorga G, Lombardia L, Herrera M, Dominguez O, Segura MF, Bonilla F, Hernando E, Munoz A (2012). MicroRNA-22 is induced by vitamin D and contributes to its antiproliferative, antimigratory and gene regulatory effects in colon cancer cells. Hum Mol Genet.

[58] Nagaraja AK, Creighton CJ, Yu Z, Zhu H, Gunaratne PH, Reid JG, Olokpa E, Itamochi H, Ueno NT, Hawkins SM, Anderson ML, Matzuk MM (2010). A link between mir-100 and FRAP1/mTOR in clear cell ovarian cancer. Mol Endocrinol.

[59] Franchina T, Amodeo V, Bronte G, Savio G, Ricciardi GR, Picciotto M, Russo A, Giordano A, Adamo V (2014). Circulating miR-22, miR-24 and miR-34a as novel predictive biomarkers to pemetrexed-based chemotherapy in advanced non-small cell lung cancer. J Cell Physiol.

[60] Pasqualini L, Bu H, Puhr M, Narisu N, Rainer J, Schlick B, Schafer G, Angelova M, Trajanoski Z, Borno ST, Schweiger MR, Fuchsberger C, Klocker H (2015). miR-22 and miR-29a are members of the androgen receptor cistrome modulating LAMC1 and Mcl-1 in prostate cancer. Mol Endocrinol.

[61] Saydam O, Shen Y, Wurdinger T, Senol O, Boke E, James MF, Tannous BA, Stemmer-Rachamimov AO, Yi M, Stephens RM, Fraefel C, Gusella JF, Krichevsky AM, Breakefield XO (2009). Downregulated microRNA-200a in meningiomas promotes tumor growth by reducing E-cadherin and activating the Wnt/beta-catenin signaling pathway. Mol Cell Biol.

[62] Wang C, Hann HW, Ye Z, Hann RS, Wan S, Ye X, Block PD, Li B, Myers RE, Wang X, Juon HS, Civan J, Chang M (2016). Prospective evidence of a circulating microRNA signature as a non-invasive marker of hepatocellular carcinoma in HBV patients. Oncotarget.

[63] Yu F, Lu Z, Chen B, Dong P, Zheng J (2015). microRNA-150: a promising novel biomarker for hepatitis B virus-related hepatocellular carcinoma. Diagn Pathol.

[64] Fayyad-Kazan H, Bitar N, Najar M, Lewalle P, Fayyad-Kazan M, Badran R, Hamade E, Daher A, Hussein N, El Dirani R, Berri F, Vanhamme L, Burny A (2013). Circulating miR-150 and miR-342 in plasma are novel potential biomarkers for acute myeloid leukemia. J Transl Med.

[65] Meyer SU, Pfaffl MW, Ulbrich SE (2010). Normalization strategies for microRNA profiling experiments: a 'normal' way to a hidden layer of complexity?. Biotechnol Lett.

[66] Andersen CL, Jensen JL, Orntoft TF (2004). Normalization of real-time quantitative reverse transcription-PCR data: a model-based variance estimation approach to identify genes suited for normalization, applied to bladder and colon cancer data sets. Cancer Res.

[67] Chou CH, Chang NW, Shrestha S, Hsu SD, Lin YL, Lee WH, Yang CD, Hong HC, Wei TY, Tu SJ, Tsai TR, Ho SY, Jian TY (2016). miRTarBase 2016: updates to the experimentally validated miRNA-target interactions database. Nucleic Acids Res.

