# Supplementary material for: Profiling of metastatic small intestine neuroendocrine tumors reveals characteristic miRNAs detectable in plasma
Source: Oncotarget. 2017 Apr 7;8(33):54331–44. doi: 10.18632/oncotarget.16908 (PMC5589584; doi:10.18632/oncotarget.16908)
Supplement: Supplementary file 1 [file oncotarget-08-54331-s001.pdf]

Profiling of metastatic small intestine neuroendocrine tumors reveals characteristic miRNAs detectable in plasma

SUPPLEMENTARY MATERIALS

Supplementary Table 1A: SINET tumors tissues utilized in small RNA sequencing

Deidentified ID	Age	Sex	Tissue specimen collected	Primary tumor	
CAR7	55.82	F	ileum	Small Bowel	primary
CAR12	71.06	M	ileum	Small Bowel	
CAR17	47.95	F	small bowel	UNK Primary	
CAR20	73.14	F	small bowel	Small Bowel	
CAR26	68.49	F	small bowel	Small Bowel	
CAR1	44.07	F	mesenteric mass	Small Bowel	metastatic
CAR3	67.61	F	mesenteric mass	Small Bowel	
CAR4	57.54	M	mesenteric mass	Small Bowel	
CAR8	57.07	M	mesenteric mass	Small Bowel	
CAR6	78.66	F	mesenteric mass	UNK Primary	
CAR21	70.86	F	mesenteric mass	Small Bowel	
CAR23	43.37	M	mesenteric mass	Small Bowel	
CAR27	60.18	M	mesenteric mass	Small Bowel	
CAR10	73.43	M	liver	Small Bowel	
CAR18c	57.96	M	liver	Small Bowel	
CAR19	66.67	F	liver	Small Bowel	
CAR11	49.90	F	liver	Small Bowel	
CAR2	57.14	F	ovary	Small Bowel	
CAR5	48.01	F	ovary	UNK Primary	

Supplementary Table 1B: Follow-up cohort for 31-miR panel interim testing

See Supplementary File 1

Supplementary Table 1C: Validation cohort for miR-21-5p, miR-22-3p, miR-29b-3p and 150-5p testing

See Supplementary File 1

Supplementary Table 2: 31 candidate miR's that were identified by combining the miR-Seq and profiling data sets

miR name	Criteria satisfied	# miRs	# miRs
hsa-miR-629-5p	miR-Seq concordance (low exp in both)	1	1
hsa-let-7a-5p	top 10 Exiqon (p<0.05), no exp in tissue	3	3
hsa-miR-10a-5p	top 10 Exiqon (p<0.05), no exp in tissue		
hsa-miR-365a-3p	top 10 Exiqon (p<0.05), no exp in tissue		
hsa-miR-144-5p	top 50 Exiqon(P<0.05), miR-Seq concordance	9	27
hsa-miR-148b-3p	top 50 Exiqon(P<0.05), miR-Seq concordance		
hsa-miR-150-5p	top 50 Exiqon(P<0.05), miR-Seq concordance		
hsa-miR-185-5p	top 50 Exiqon(P<0.05), miR-Seq concordance		
hsa-miR-19a-3p	top 50 Exiqon(P<0.05), miR-Seq concordance		
hsa-miR-222-3p	top 50 Exiqon(P<0.05), miR-Seq concordance		
hsa-miR-22-3p	top 50 Exiqon(P<0.05), miR-Seq concordance		
hsa-miR-29a-3p	top 50 Exiqon(P<0.05), miR-Seq concordance		
hsa-miR-423-5p	top 50 Exiqon(P<0.05), miR-Seq concordance		
hsa-miR-107	top 50 Exiqon, miR-Seq concordance	18	
hsa-let-7g-5p	top 50 Exiqon, miR-Seq concordance		
hsa-miR-122-5p	top 50 Exiqon, miR-Seq concordance		
hsa-miR-126-3p	top 50 Exiqon, miR-Seq concordance		
hsa-miR-143-3p	top 50 Exiqon, miR-Seq concordance		
hsa-miR-145-5p	top 50 Exiqon, miR-Seq concordance		
hsa-miR-146a-5p	top 50 Exiqon, miR-Seq concordance		
hsa-miR-151a-3p	top 50 Exiqon, miR-Seq concordance		
hsa-miR-15b-5p	top 50 Exiqon, miR-Seq concordance		
hsa-miR-19b-3p	top 50 Exiqon, miR-Seq concordance		
hsa-miR-20a-5p	top 50 Exiqon, miR-Seq concordance		
hsa-miR-21-5p	top 50 Exiqon, miR-Seq concordance		
hsa-miR-221-3p	top 50 Exiqon, miR-Seq concordance		
hsa-miR-29b-3p	top 50 Exiqon, miR-Seq concordance		
hsa-miR-29c-3p	top 50 Exiqon, miR-Seq concordance		
hsa-miR-425-5p	top 50 Exiqon, miR-Seq concordance		
hsa-miR-497-5p	top 50 Exiqon, miR-Seq concordance		
hsa-miR-93-5p	top 50 Exiqon, miR-Seq concordance		
	Total	31	31
		**Subtotal [%]**	**87%**

Supplementary Table 3: Pearson correlation coefficients for comparison between miR-21-5p, miR-22-3p and miR-150-5p

Pearson correlation coefficients, N = 111<br>Prob > |r| under H0: Rho=0				
	**std_targ_21**	**std_targ_22**	**std_targ_29b**	**std_targ_150**
std_targ_21	1	0.41033<br><.0001	-0.12393<br>0.195	-0.02923<br>0.7607
std_targ_22	0.41033<br><.0001	1	0.05613<br>0.5585	-0.46611<br><.0001
std_targ_29b	-0.12393<br>0.195	0.05613<br>0.5585	1	0.13<br>0.1739
std_targ_150	-0.02923<br>0.7607	-0.46611<br><.0001	0.13<br>0.1739	1

Supplementary Table 4: Target genes regulated by miR-21-5p (strong evidence - reporter assay, qRTPCR, WB) using MiRTarBase 6.0

See Supplementary File 2
