# Supplementary material for: Profiling of metastatic small intestine neuroendocrine tumors reveals characteristic miRNAs detectable in plasma
Source: Oncotarget. 2017 Apr 7;8(33):54331–44. doi: 10.18632/oncotarget.16908 (PMC5589584; doi:10.18632/oncotarget.16908)
Supplement: Supplementary file 2 [file oncotarget-08-54331-s002.docx]

| Supplementary Table 1B: Follow-up cohort for 31-miR panel interim testing | | | | | |  |
| --- | --- | --- | --- | --- | --- | --- |
| **ID** | **DOB** | **Gender** | **Race** | **Primary site** | **Sample period** | **Differentiation** |
| NEUB1023-3 | 10/11/1940 | M | White | Small Intestine | Mets on treatment | Well |
| NEUB1179-3 | 3/26/1954 | M | White | Small Intestine | Mets treatment naive | Well |
| NEUB1200-3 | 9/6/1955 | F | White | Small Intestine | Primary only on treatment | Well |
| NEUB1266-3 | 2/15/1950 | F | White | Small Intestine | Mets on treatment | Well |
| NEUB1434-3 | 6/7/1948 | M | White | Small Intestine | Mets S/P treatment | Well |
| NEUB1509-3 | 10/15/1957 | M | White | Small Intestine | Mets treatment naive | Well |
| NEUB1515-3 | 5/28/1935 | F | White | Small Intestine | Mets on treatment | Well |
| NEUB1584-3 | 12/22/1939 | F | White | Small Intestine | Mets treatment naive | Well |
| NEUB1610-3 | 4/20/1945 | F | White | Small Intestine | Mets treatment naive | Well |
| NEUB1787-3 | 9/7/1948 | M | White | Small Intestine | Mets on treatment | Well |
| NEUB1841-3 | 6/6/1941 | F | White | Small Intestine | Mets S/P treatment | Well |
| NEUB1895-3 | 11/13/1944 | M | White | Small Intestine | Mets treatment naive | Well |
| NEUB1971-3 | 12/21/1966 | M | Unknown | Small Intestine | Mets on treatment | Well |
| NEUB2046-3 | 10/20/1943 | M | White | Small Intestine | Mets on treatment | Well |
| NEUB2076-3 | 6/6/1947 | M | White | Small Intestine | Mets on treatment | Well |
| NEUB2216-3 | 12/10/1954 | M | White | Small Intestine | Mets on treatment | Well |
| NEUB2234-3 | 11/14/1950 | M | White | Small Intestine | Mets treatment naive | Well |
| NEUB2305-3 | 8/28/1948 | F | Black or African American | Small Intestine | Mets on treatment | Well |
| NEUB2326-3 | 7/6/1942 | F | White | Small Intestine | Mets on treatment | Well |
| NEUB2341-3 | 4/26/1967 | F | White | Small Intestine | Mets on treatment | Well |
| NEUB2396-3 | 5/13/1948 | F | White | Small Intestine | Unknown | Well |
| NEUB2480-3 | 8/16/1941 | M | White | Small Intestine | Mets on treatment | Well |
| NEUB2486-3 | 2/18/1949 | F | Unknown | Small Intestine | Mets on treatment | Well |
| NEUB2583-3 | 2/25/1949 | M | White | Small Intestine | NED treatment naive | Well |
| NEUB2643-3 | 8/7/1945 | M | White | Small Intestine | Mets on treatment | Well |
| NEUB2930-3 | 12/13/1946 | F | White | Small Intestine | Mets on treatment | Well |
| NEUB3047-3 | 4/9/1957 | F | White | Small Intestine | Mets treatment naive | Well |
| NEUB3266-3 | 4/1/1959 | M | White | Small Intestine | Mets treatment naive | Well |
| NEUB3332-3 | 7/7/1951 | M | White | Small Intestine | Mets on treatment | Well |
| NEUB3382-3 | 9/19/1948 | F | White | Small Intestine | Unknown | Well |
| NEUB3435-3 | 4/5/1945 | F | White | Small Intestine | Mets on treatment | Poor |
| NEUB3441-3 | 5/20/1946 | F | White | Small Intestine | Mets on treatment | Well |
| NEUB3513-3 | 7/28/1952 | F | White | Small Intestine | Mets on treatment | Well |
| NEUB3622-3 | 9/12/1950 | F | Unknown | Small Intestine | Mets on treatment | Well |
| NEUB3625-3 | 5/19/1949 | F | White | Small Intestine | Mets on treatment | Well |
| NEUB3774-3 | 8/1/1947 | M | White | Small Intestine | Mets treatment naive | Well |
| NEUB3829-3 | 6/26/1965 | F | White | Small Intestine | Mets treatment naive | Well |
| NEUB3921-3 | 9/28/1960 | M | White | Small Intestine | Mets treatment naive | Well |
| NEUB3981-3 | 8/6/1946 | F | White | Small Intestine | Mets on treatment | Well |
| NEUB4038-3 | 7/30/1958 | F | White | Small Intestine | Mets on treatment | Well |
| CTLB0029-3 | 2/12/1938 | M |  |  |  |  |
| CTLB0074-3 | 1/12/1946 | M |  |  |  |  |
| CTLB0128-3 | 8/9/1941 | M |  |  |  |  |
| CTLB0146-3 | 3/29/1942 | M |  |  |  |  |
| CTLB0149-3 | 3/2/1941 | M |  |  |  |  |
| CTLB0188-4 | 12/23/1938 | F |  |  |  |  |
| CTLB0239-3 | 7/4/1942 | F |  |  |  |  |
| CTLB0272-3 | 12/10/1939 | F |  |  |  |  |
| CTLB0299-3 | 8/27/1941 | M |  |  |  |  |
| CTLB0317-4 | 3/13/1935 | F |  |  |  |  |
| CTLB0341-3 | 9/29/1946 | M |  |  |  |  |
| CTLB0371-3 | 4/25/1956 | F |  |  |  |  |
| CTLB0386-3 | 9/13/1955 | M |  |  |  |  |
| CTLB0389-3 | 9/25/1950 | F |  |  |  |  |
| CTLB0401-3 | 2/9/1955 | M |  |  |  |  |
| CTLB0434-3 | 12/2/1940 | F |  |  |  |  |
| CTLB0464-3 | 1/2/1949 | F |  |  |  |  |
| CTLB0482-3 | 11/27/1954 | F |  |  |  |  |
| CTLB0494-3 | 10/7/1947 | M |  |  |  |  |
| CTLB0554-3 | 12/11/1942 | F |  |  |  |  |
| CTLB0557-3 | 3/10/1941 | F |  |  |  |  |
| CTLB0590-3 | 12/13/1946 | F |  |  |  |  |
| CTLB0611-3 | 11/27/1943 | F |  |  |  |  |
| CTLB0632-3 | 1/14/1951 | M |  |  |  |  |
| CTLB0650-3 | 4/14/1949 | F |  |  |  |  |
| CTLB0695-3 | 3/22/1949 | F |  |  |  |  |
| CTLB0721-3 | 9/19/1949 | F |  |  |  |  |
| CTLB0727-3 | 4/8/1968 | F |  |  |  |  |
| CTLB0784-3 | 7/8/1969 | M |  |  |  |  |
| CTLB0790-3 | 7/12/1958 | M |  |  |  |  |
| CTLB0841-3 | 6/26/1949 | M |  |  |  |  |
| CTLB0901-3 | 9/7/1952 | M |  |  |  |  |
| CTLB0919-3 | 10/5/1963 | F |  |  |  |  |
| CTLB0922-3 | 6/10/1961 | M |  |  |  |  |
| CTLB1000-3 | 11/7/1949 | F |  |  |  |  |
| CTLB1051-3 | 8/4/1948 | F |  |  |  |  |
| CTLB1105-3 | 5/29/1946 | M |  |  |  |  |
| CTLB1111-3 | 2/24/1949 | F |  |  |  |  |
| CTLB1135-3 | 3/1/1958 | M |  |  |  |  |
| CTLB1183-3 | 12/16/1957 | F |  |  |  |  |
|  |  |  |  |  |  |  |
| NEUB: SINET Pt |  |  |  |  |  |  |
| CTLB: Healthy Control | |  |  |  |  |  |

| Supplementary Table 1C: Validation cohort for miR-21-5p, miR-22-3p, miR-29b-3p and 150-5p testing | | | | | | | |  |  |  |
| --- | --- | --- | --- | --- | --- | --- | --- | --- | --- | --- |
| **ID** | **Hemolysis__Excluded** | **DOB** | **Gender** | **Race** | **Primary site** | **Differentiation** | **Treatment Status** | **Sample_period** | **CgA_Value*** | **2x elevated level** |
| NEUB0156 | Include | 7/26/1935 | F | Unknown or Not Reported | Small Intestine | WELL | Octreotide | Mets on treatment | 205 | Yes |
| NEUB0165 | Exclude | 1/9/1936 | F | White | Small Intestine | WELL | Octreotide | Mets on treatment | 55 | Yes |
| NEUB0168 | Exclude | 2/2/1939 | F | White | Small Intestine | WELL | Octreotide | Mets on treatment | 18.1 | Unknown |
| NEUB0177 | Exclude | 9/19/1933 | F | White | Small Intestine | WELL | Octreotide | Mets on treatment | 37.7 | No |
| NEUB0192 | Include | 4/25/1949 | M | White | Small Intestine | WELL | Octreotide | Mets on treatment | 2930 | Yes |
| NEUB0264 | Include | 10/13/1930 | M | White | Small Intestine | WELL | Octreotide | Mets S/P treatment | 181 | Yes |
| NEUB0297 | Include | 10/21/1931 | F | White | Small Intestine | WELL | Octreotide | Mets on treatment | 24.8 | No |
| NEUB0326 | Include | 9/27/1945 | M | White | Small Intestine | WELL | Octreotide | Mets on treatment | 1320 | Yes |
| NEUB0356 | Include | 2/16/1944 | M | White | Small Intestine | WELL | Octreotide | Mets on treatment | 2750 | Yes |
| NEUB0374 | Include | 4/5/1960 | M | Black or African American | Small Intestine | WELL | Octreotide | Mets on treatment | 68 | Yes |
| NEUB0395 | Include | 1/12/1930 | F | White | Small Intestine | WELL | Octreotide | Mets treatment naive | 187 | Unknown |
| NEUB0419 | Include | 5/5/1940 | M | White | Small Intestine | WELL | Octreotide | Mets on treatment | 15.9 | No |
| NEUB0436 | Include | 12/1/1945 | F | Unknown or Not Reported | Small Intestine | WELL | treatment naïve | Mets treatment naive |  | N/A |
| NEUB0457 | Include | 7/11/1969 | M | White | Small Intestine | WELL | treatment naïve | Mets treatment naive | 1580 | Yes |
| NEUB0505 | Include | 2/7/1931 | M | White | Small Intestine | WELL | Octreotide | Mets treatment naive | 300 | Yes |
| NEUB0526 | Include | 12/4/1940 | M | Black or African American | Small Intestine | WELL | treatment naïve | Mets treatment naive | 40 | Yes |
| NEUB0553 | Include | 3/4/1962 | F | White | Small Intestine | UNK | Bevacizumab/Temozolomide | Mets on treatment | 910 | Yes |
| NEUB0559 | Include | 7/6/1968 | M | White | Small Intestine | WELL | Octreotide | Mets treatment naive | 16.4 | No |
| NEUB0565 | Exclude | 7/31/1949 | M | White | Small Intestine | WELL | Bevacizumab/Temozolomide | Mets treatment naive | 45 | Unknown |
| NEUB0571 | Include | 7/8/1935 | M | White | Small Intestine | WELL | Octreotide | Mets treatment naive | 410 | Yes |
| NEUB0619 | Include | 3/17/1948 | F | Unknown or Not Reported | Small Intestine | WELL | Octreotide | Mets treatment naive | 332 | Yes |
| NEUB0631 | Include | 12/7/1930 | F | White | Small Intestine | WELL | Octreotide | Mets on treatment |  | N/A |
| NEUB0697 | Include | 12/24/1951 | M | White | Small Intestine | WELL | Octreotide | Mets on treatment |  | N/A |
| NEUB0774 | Include | 11/9/1957 | F | White | Small Intestine | WELL | treatment naïve | Mets treatment naive |  | N/A |
| NEUB0783 | Include | 2/8/1933 | F | White | Small Intestine | WELL | Octreotide | Mets treatment naive | 570 | Yes |
| NEUB0792 | Include | 4/10/1948 | M | White | Small Intestine | WELL | Octreotide | Mets treatment naive | 97 | No |
| NEUB0798 | Include | 9/15/1966 | M | White | Small Intestine | WELL | Octreotide | Mets treatment naive | 5950 | Yes |
| NEUB0843 | Include | 3/1/1943 | M | White | Small Intestine | WELL | Octreotide | Mets treatment naive | 96 | No |
| NEUB0897 | Include | 7/16/1948 | F | White | Small Intestine | WELL | Octreotide | Mets on treatment | 291 | No |
| NEUB0912 | Include | 3/24/1973 | F | White | Small Intestine | WELL | treatment naïve | Mets treatment naive |  | Unknown |
| NEUB0921 | Include | 7/1/1927 | M | White | Small Intestine | WELL | Octreotide | Mets treatment naive | 41 | Yes |
| NEUB0960 | Include | 1/8/1922 | M | White | Small Intestine | WELL | Octreotide | Mets treatment naive |  | N/A |
| NEUB0969 | Include | 2/3/1937 | M | White | Small Intestine | WELL | treatment naïve | Mets treatment naive | 268 | Yes |
| NEUB0993 | Include | 8/25/1956 | M | White | Small Intestine | WELL | treatment naïve | Mets treatment naive |  | N/A |
| NEUB1020 | Include | 12/12/1937 | F | White | Small Intestine | WELL | treatment naïve | Mets treatment naive | 41.5 | Yes |
| NEUB1092 | Include | 5/20/1934 | M | White | Small Intestine | WELL | Octreotide | Mets on treatment |  | N/A |
| NEUB1104 | Include | 1/5/1943 | F | White | Small Intestine | WELL | Octreotide | Mets treatment naive | 1925 | Unknown |
| NEUB1116 | Include | 4/9/1938 | M | White | Small Intestine | WELL | Octreotide | Mets S/P treatment |  | N/A |
| NEUB1173 | Include | 2/16/1937 | F | White | Small Intestine | WELL | Octreotide | Mets on treatment | 377 | Unknown |
| NEUB1194 | Include | 8/20/1934 | F | White | Small Intestine | WELL | Octreotide | Mets S/P treatment |  | N/A |
| NEUB1218 | Include | 9/4/1943 | M | White | Small Intestine | WELL | treatment naïve | Mets S/P treatment |  | N/A |
| NEUB1293 | Include | 7/19/1937 | M | White | Small Intestine | WELL | Octreotide | Mets on treatment | 580 | Unknown |
| NEUB1368 | Include | 3/19/1946 | F | White | Small Intestine | WELL | Octreotide | Mets on treatment | 60 | No |
| NEUB1386 | Include | 12/10/1952 | F | White | Small Intestine | WELL | Octreotide | Mets on treatment | 2670 | Yes |
| NEUB1401 | Include | 1/16/1948 | M | White | Small Intestine | WELL | Octreotide | Mets treatment naive | 2101 | Unknown |
| NEUB1410 | Include | 4/8/1955 | M | White | Small Intestine | WELL | Octreotide | Mets S/P treatment | 24.1 | Unknown |
| NEUB1560 | Include | 6/30/1952 | M | White | Small Intestine | WELL | Interferon | Mets on treatment |  | N/A |
| NEUB1566 | Include | 2/13/1937 | F | White | Small Intestine | WELL | Octreotide | Mets on treatment |  | N/A |
| NEUB1596 | Include | 9/17/1963 | M | White | Small Intestine | WELL | LX-1606 | Mets treatment naive | 80 | No |
| NEUB1616 | Include | 11/14/1947 | M | White | Small Intestine | WELL | Octreotide | Mets on treatment | 452.8 | Yes |
| NEUB1667 | Include | 11/27/1929 | M | White | Small Intestine | WELL | treatment naïve | Mets treatment naive | 1845 | Unknown |
| NEUB1670 | Include | 4/25/1929 | F | White | Small Intestine | UNK | Octreotide | Mets on treatment | 327 | Yes |
| NEUB1691 | Include | 12/22/1944 | F | Black or African American | Small Intestine | WELL | Octreotide | Mets treatment naive | 125 | No |
| NEUB1766 | Include | 11/8/1948 | F | White | Small Intestine | WELL | Octreotide | Mets on treatment | 4000 | Yes |
| NEUB1883 | Include | 5/1/1939 | M | White | Small Intestine | WELL | Octreotide | Mets treatment naive | 435 | Yes |
| NEUB1938 | Include | 7/17/1936 | F | White | Small Intestine | WELL | Octreotide | Mets on treatment | 1470 | Yes |
| NEUB1968 | Include | 3/15/1934 | F | White | Small Intestine | WELL | Octreotide | Mets treatment naive | 234 | Yes |
| NEUB1992 | Include | 3/14/1933 | F | White | Small Intestine | WELL | treatment naïve | Mets treatment naive | 144.5 | Unknown |
| NEUB1998 | Include | 4/29/1951 | M | Black or African American | Small Intestine | WELL | Etoposide/Carboplatin/Paclitaxel | Mets S/P treatment | 134 | Unknown |
| NEUB2043 | Include | 2/1/1963 | F | White | Small Intestine | WELL | Octreotide | Mets on treatment | 58 | No |
| NEUB2067 | Include | 1/18/1978 | F | Black or African American | Small Intestine | WELL | treatment naïve | Mets treatment naive |  | N/A |
| NEUB2126 | Include | 1/9/1946 | F | White | Small Intestine | WELL | Octreotide | Mets treatment naive | 260 | Yes |
| NEUB2135 | Include | 2/6/1964 | F | White | Small Intestine | WELL | Octreotide | Mets on treatment |  | N/A |
| NEUB2153 | Include | 12/31/1955 | M | White | Small Intestine | WELL | Octreotide | Mets treatment naive | 9000 | Yes |
| NEUB2180 | Include | 10/12/1932 | F | White | Small Intestine | WELL | Octreotide | Mets on treatment | 3600 | Yes |
| NEUB2225 | Include | 7/9/1928 | M | Black or African American | Small Intestine | WELL | treatment naïve | Mets treatment naive | 1795 | Yes |
| NEUB2240 | Include | 12/19/1936 | F | White | Small Intestine | WELL | Octreotide | Mets on treatment | 541.6 | Yes |
| NEUB2246 | Include | 1/19/1935 | M | White | Small Intestine | UNK | treatment naïve | Mets treatment naive | 244 | Yes |
| NEUB2347 | Include | 12/26/1957 | F | White | Small Intestine | WELL | 5-Fluorouracil/Leucovorin | Mets S/P treatment | 930 | Yes |
| NEUB2375 | Include | 9/27/1952 | F | White | Small Intestine | WELL | treatment naïve | Mets treatment naive | 145 | No |
| NEUB2453 | Include | 8/8/1945 | F | White | Small Intestine | WELL | Octreotide | Mets treatment naive | 337 | Yes |
| NEUB2477 | Include | 3/26/1943 | F | White | Small Intestine | WELL | Octreotide | Mets on treatment | 338 | Yes |
| NEUB2510 | Include | 9/24/1929 | F | White | Small Intestine | WELL | Octreotide | Mets on treatment | 3760 | Yes |
| NEUB2534 | Include | 4/10/1942 | M | White | Small Intestine | WELL | Octreotide | Mets on treatment | 285 | Unknown |
| NEUB2540 | Include | 5/12/1956 | M | White | Small Intestine | WELL | 5-Fluorouracil/Streptozocin | Mets S/P treatment | 2730 | Yes |
| NEUB2561 | Include | 12/17/1939 | F | White | Small Intestine | WELL | Octreotide | Mets on treatment | 645 | Yes |
| NEUB2601 | Include | 9/16/1965 | F | White | Small Intestine | WELL | treatment naïve | Mets treatment naive |  | N/A |
| NEUB2613 | Include | 10/23/1962 | M | White | Small Intestine | WELL | Octreotide | Mets on treatment | 89 | No |
| NEUB2622 | Include | 8/14/1929 | M | White | Small Intestine | WELL | treatment naïve | Mets treatment naive |  | N/A |
| NEUB2655 | Include | 3/30/1962 | F | White | Small Intestine | WELL | Octreotide | Mets on treatment | 4600 | Yes |
| NEUB2703 | Include | 4/29/1947 | M | White | Small Intestine | WELL | Octreotide | Mets on treatment | 3140 | Yes |
| NEUB2754 | Include | 9/21/1945 | M | White | Small Intestine | WELL | treatment naïve | Mets S/P treatment | 267 | Yes |
| NEUB2787 | Include | 11/5/1954 | M | White | Small Intestine | WELL | Octreotide | Mets on treatment | 30.5 | No |
| NEUB2805 | Include | 8/25/1942 | F | White | Small Intestine | WELL | Octreotide | Mets S/P treatment | 115.4 | Yes |
| NEUB2811 | Include | 5/9/1958 | M | White | Small Intestine | WELL | Octreotide | Mets on treatment | 11335 | Yes |
| NEUB2840 | Include | 10/12/1981 | M | White | Small Intestine | WELL | treatment naïve | Mets S/P treatment | 3.2 | Unknown |
| NEUB2891 | Include | 10/12/1946 | M | Black or African American | Small Intestine | WELL | Octreotide | Mets treatment naive | 805 | Yes |
| NEUB2921 | Include | 4/14/1966 | M | White | Small Intestine | WELL | treatment naïve | Mets treatment naive |  | N/A |
| NEUB2948 | Include | 9/28/1946 | F | White | Small Intestine | WELL | Octreotide | Mets on treatment | 156 | No |
| NEUB2996 | Include | 11/15/1941 | M | White | Small Intestine | WELL | Octreotide | Mets on treatment | 0.7 | Unknown |
| NEUB3044 | Include | 5/30/1962 | F | White | Small Intestine | UNK | Octreotide | Mets on treatment | 1268 | Unknown |
| NEUB3059 | Include | 2/8/1952 | F | White | Small Intestine | WELL | Octreotide | Mets treatment naive |  | N/A |
| NEUB3089 | Include | 1/7/1948 | M | Native Hawaiian or Other Pacific Islander | Small Intestine | WELL | treatment naïve | Mets S/P treatment | 233 | Yes |
| NEUB3143 | Include | 3/1/1943 | F | White | Small Intestine | WELL | Octreotide | Mets treatment naive | 435 | Yes |
| NEUB3146 | Include | 4/16/1944 | F | White | Small Intestine | WELL | treatment naïve | Mets treatment naive | 23 | No |
| NEUB3272 | Include | 2/9/1938 | F | Unknown or Not Reported | Small Intestine | WELL | Octreotide | Mets on treatment | 4480 | Yes |
| NEUB3311 | Include | 5/9/1947 | F | White | Small Intestine | WELL | Octreotide | Mets on treatment | 15750 | Yes |
| NEUB3367 | Include | 12/5/1936 | M | White | Small Intestine | WELL | Octreotide | Mets on treatment | 331 | Yes |
| NEUB3462 | Include | 6/8/1959 | F | White | Small Intestine | WELL | Octreotide | Mets on treatment | 490 | Yes |
| NEUB3495 | Include | 10/24/1940 | M | White | Small Intestine | WELL | Octreotide | Mets treatment naive | 242 | Yes |
| NEUB3507 | Include | 9/25/1979 | F | White | Small Intestine | WELL | Octreotide | Mets on treatment | 94 | No |
| NEUB3533 | Include | 10/15/1936 | M | White | Small Intestine | WELL | Octreotide | Mets treatment naive | 2770 | Yes |
| NEUB3646 | Include | 11/2/1942 | M | White | Small Intestine | WELL | treatment naïve | Mets S/P treatment |  | N/A |
| NEUB3685 | Include | 11/5/1955 | F | White | Small Intestine | WELL | Octreotide | Mets on treatment | 364 | Yes |
| NEUB3688 | Include | 11/6/1956 | F | White | Small Intestine | WELL | Octreotide | Mets treatment naive | 16.2 | Yes |
| NEUB3712 | Include | 12/4/1940 | M | White | Small Intestine | POOR | treatment naïve | Mets treatment naive |  | N/A |
| NEUB3718 | Include | 4/22/1938 | M | White | Small Intestine | WELL | treatment naïve | Mets treatment naive | 201 | Yes |
| NEUB3724 | Include | 8/31/1942 | F | White | Small Intestine | WELL | Octreotide | Mets on treatment | 830 | Unknown |
| NEUB3862 | Exclude | 2/20/1944 | M | Unknown or Not Reported | Small Intestine | WELL | Octreotide | Mets S/P treatment | 40 | No |
| NEUB3871 | Exclude | 3/19/1959 | F | White | Small Intestine | WELL | Octreotide | Mets on treatment | 236 | Yes |
| NEUB3877 | Exclude | 6/14/1960 | M | White | Small Intestine | WELL | Octreotide | Mets on treatment | 36 | No |
| NEUB4023 | Include | 6/13/1938 | F | White | Small Intestine | WELL | Octreotide | Mets treatment naive | 715 | Yes |
| NEUB4059 | Include | 12/14/1961 | F | White | Small Intestine | UNK | Octreotide | Mets on treatment | 90 | Unknown |
| NEUB4070 | Include | 9/22/1950 | M | White | Small Intestine | WELL | Octreotide | Mets treatment naive | 140 | Yes |
| NEUB4082 | Include | 1/7/1956 | M | White | Small Intestine | WELL | Octreotide | Mets treatment naive |  | N/A |
| NEUB4099 | Include | 6/18/1943 | M | White | Small Intestine | WELL | Octreotide | Mets on treatment | 93 | Yes |
| NEUB4171 | Exclude | 12/10/1948 | M | White | Small Intestine | WELL | Octreotide | Mets on treatment | 180 | Yes |
| NEUB4195 | Include | 4/1/1953 | M | White | Small Intestine | WELL | Octreotide | Mets treatment naive | 62 | No |
| NEUB4230 | Exclude | 1/9/1963 | F | Unknown or Not Reported | Small Intestine | WELL | treatment naïve | Mets treatment naive | 112 | Yes |
| NEUB4310 | Include | 2/26/1952 | F | White | Small Intestine | WELL | treatment naïve | Mets treatment naive | 68 | No |
| CTLB0005-4 | Include | 11/27/1973 | F |  |  |  |  |  |  |  |
| CTLB0008-4 | Include | 2/21/1957 | F |  |  |  |  |  |  |  |
| CTLB0017-4 | Include | 5/24/1947 | M |  |  |  |  |  |  |  |
| CTLB0020-4 | Include | 6/9/1961 | M |  |  |  |  |  |  |  |
| CTLB0023-4 | Include | 8/2/1934 | F |  |  |  |  |  |  |  |
| CTLB0056-4 | Include | 8/15/1954 | M |  |  |  |  |  |  |  |
| CTLB0062-4 | Include | 9/23/1932 | F |  |  |  |  |  |  |  |
| CTLB0071-4 | Include | 11/23/1947 | M |  |  |  |  |  |  |  |
| CTLB0077-4 | Include | 4/6/1958 | F |  |  |  |  |  |  |  |
| CTLB0083-4 | Include | 12/14/1946 | M |  |  |  |  |  |  |  |
| CTLB0092-4 | Include | 7/29/1950 | F |  |  |  |  |  |  |  |
| CTLB0104-4 | Include | 1/2/1939 | M |  |  |  |  |  |  |  |
| CTLB0117-5 | Include | 3/1/1925 | F |  |  |  |  |  |  |  |
| CTLB0122-4 | Include | 5/28/1922 | F |  |  |  |  |  |  |  |
| CTLB0137-5 | Include | 11/15/1972 | F |  |  |  |  |  |  |  |
| CTLB0152-4 | Include | 12/3/1940 | F |  |  |  |  |  |  |  |
| CTLB0155-4 | Include | 3/2/1928 | M |  |  |  |  |  |  |  |
| CTLB0176-4 | Include | 9/23/1952 | M |  |  |  |  |  |  |  |
| CTLB0179-4 | Include | 10/9/1937 | F |  |  |  |  |  |  |  |
| CTLB0182-4 | Include | 12/17/1952 | M |  |  |  |  |  |  |  |
| CTLB0200-4 | Include | 3/8/1953 | M |  |  |  |  |  |  |  |
| CTLB0209-5 | Include | 2/28/1926 | M |  |  |  |  |  |  |  |
| CTLB0227-4 | Include | 3/28/1957 | M |  |  |  |  |  |  |  |
| CTLB0245-4 | Include | 7/19/1944 | F |  |  |  |  |  |  |  |
| CTLB0254-4 | Include | 11/24/1940 | M |  |  |  |  |  |  |  |
| CTLB0275-4 | Include | 7/8/1943 | M |  |  |  |  |  |  |  |
| CTLB0314-4 | Include | 4/25/1937 | F |  |  |  |  |  |  |  |
| CTLB0326-4 | Include | 9/20/1955 | M |  |  |  |  |  |  |  |
| CTLB0332-4 | Include | 2/18/1936 | M |  |  |  |  |  |  |  |
| CTLB0338-4 | Include | 12/9/1961 | F |  |  |  |  |  |  |  |
| CTLB0362-4 | Include | 11/18/1955 | F |  |  |  |  |  |  |  |
| CTLB0365-4 | Include | 6/26/1949 | M |  |  |  |  |  |  |  |
| CTLB0380-4 | Include | 3/8/1937 | F |  |  |  |  |  |  |  |
| CTLB0383-5 | Include | 7/9/1950 | M |  |  |  |  |  |  |  |
| CTLB0392-5 | Include | 12/24/1955 | M |  |  |  |  |  |  |  |
| CTLB0395-4 | Include | 12/13/1934 | F |  |  |  |  |  |  |  |
| CTLB0398-4 | Include | 7/29/1932 | M |  |  |  |  |  |  |  |
| CTLB0404-4 | Include | 3/18/1957 | M |  |  |  |  |  |  |  |
| CTLB0407-5 | Include | 4/5/1943 | F |  |  |  |  |  |  |  |
| CTLB0413-4 | Include | 4/12/1957 | M |  |  |  |  |  |  |  |
| CTLB0419-4 | Include | 1/9/1947 | M |  |  |  |  |  |  |  |
| CTLB0428-4 | Include | 3/4/1938 | F |  |  |  |  |  |  |  |
| CTLB0440-4 | Include | 11/12/1937 | M |  |  |  |  |  |  |  |
| CTLB0458-4 | Include | 4/18/1951 | F |  |  |  |  |  |  |  |
| CTLB0467-4 | Include | 12/23/1963 | M |  |  |  |  |  |  |  |
| CTLB0470-4 | Include | 7/2/1940 | F |  |  |  |  |  |  |  |
| CTLB0515-4 | Include | 3/20/1957 | F |  |  |  |  |  |  |  |
| CTLB0527-4 | Include | 11/7/1942 | M |  |  |  |  |  |  |  |
| CTLB0530-4 | Include | 5/20/1972 | M |  |  |  |  |  |  |  |
| CTLB0539-4 | Include | 4/27/1937 | F |  |  |  |  |  |  |  |
| CTLB0563-4 | Include | 5/4/1945 | F |  |  |  |  |  |  |  |
| CTLB0566-4 | Include | 5/18/1940 | F |  |  |  |  |  |  |  |
| CTLB0569-4 | Include | 7/4/1955 | M |  |  |  |  |  |  |  |
| CTLB0575-4 | Include | 9/1/1937 | F |  |  |  |  |  |  |  |
| CTLB0578-4 | Include | 9/20/1940 | F |  |  |  |  |  |  |  |
| CTLB0581-4 | Include | 2/18/1954 | M |  |  |  |  |  |  |  |
| CTLB0596-4 | Include | 8/26/1948 | M |  |  |  |  |  |  |  |
| CTLB0614-4 | Include | 12/13/1970 | M |  |  |  |  |  |  |  |
| CTLB0620-4 | Include | 9/20/1955 | F |  |  |  |  |  |  |  |
| CTLB0623-4 | Include | 9/2/1934 | F |  |  |  |  |  |  |  |
| CTLB0641-4 | Include | 10/23/1981 | M |  |  |  |  |  |  |  |
| CTLB0647-4 | Include | 4/7/1952 | M |  |  |  |  |  |  |  |
| CTLB0653-4 | Include | 11/5/1944 | F |  |  |  |  |  |  |  |
| CTLB0656-4 | Include | 3/29/1954 | M |  |  |  |  |  |  |  |
| CTLB0662-4 | Include | 3/14/1941 | F |  |  |  |  |  |  |  |
| CTLB0665-4 | Include | 12/21/1951 | M |  |  |  |  |  |  |  |
| CTLB0668-4 | Include | 11/17/1941 | F |  |  |  |  |  |  |  |
| CTLB0707-4 | Include | 5/29/1949 | F |  |  |  |  |  |  |  |
| CTLB0724-4 | Include | 3/26/1966 | F |  |  |  |  |  |  |  |
| CTLB0748-4 | Include | 6/3/1948 | M |  |  |  |  |  |  |  |
| CTLB0751-4 | Include | 8/27/1956 | F |  |  |  |  |  |  |  |
| CTLB0754-4 | Include | 6/18/1953 | F |  |  |  |  |  |  |  |
| CTLB0757-4 | Include | 12/23/1959 | M |  |  |  |  |  |  |  |
| CTLB0781-4 | Include | 8/6/1960 | M |  |  |  |  |  |  |  |
| CTLB0811-4 | Include | 11/5/1936 | M |  |  |  |  |  |  |  |
| CTLB0814-4 | Include | 8/15/1959 | M |  |  |  |  |  |  |  |
| CTLB0823-4 | Include | 10/1/1938 | M |  |  |  |  |  |  |  |
| CTLB0826-4 | Include | 7/10/1941 | F |  |  |  |  |  |  |  |
| CTLB0832-4 | Include | 5/25/1968 | F |  |  |  |  |  |  |  |
| CTLB0838-4 | Include | 4/24/1940 | F |  |  |  |  |  |  |  |
| CTLB0868-4 | Include | 10/2/1957 | M |  |  |  |  |  |  |  |
| CTLB0883-4 | Include | 5/15/1946 | F |  |  |  |  |  |  |  |
| CTLB0895-4 | Include | 7/21/1953 | M |  |  |  |  |  |  |  |
| CTLB0913-4 | Include | 1/30/1956 | M |  |  |  |  |  |  |  |
| CTLB0937-4 | Include | 5/19/1954 | F |  |  |  |  |  |  |  |
| CTLB0952-4 | Include | 9/25/1954 | F |  |  |  |  |  |  |  |
| CTLB0958-4 | Include | 4/4/1943 | F |  |  |  |  |  |  |  |
| CTLB0961-4 | Include | 3/6/1976 | M |  |  |  |  |  |  |  |
| CTLB0982-4 | Include | 2/14/1945 | M |  |  |  |  |  |  |  |
| CTLB0985-4 | Include | 5/1/1941 | F |  |  |  |  |  |  |  |
| CTLB0991-4 | Include | 4/13/1947 | M |  |  |  |  |  |  |  |
| CTLB1006-4 | Include | 10/23/1952 | F |  |  |  |  |  |  |  |
| CTLB1015-4 | Include | 10/3/1949 | M |  |  |  |  |  |  |  |
| CTLB1024-4 | Include | 7/24/1957 | M |  |  |  |  |  |  |  |
| CTLB1027-4 | Include | 5/30/1946 | F |  |  |  |  |  |  |  |
| CTLB1045-4 | Include | 11/27/1947 | F |  |  |  |  |  |  |  |
| CTLB1054-4 | Include | 5/6/1952 | F |  |  |  |  |  |  |  |
| CTLB1057-4 | Include | 11/29/1946 | M |  |  |  |  |  |  |  |
| CTLB1066-4 | Include | 10/7/1947 | M |  |  |  |  |  |  |  |
| CTLB1078-4 | Include | 8/26/1950 | M |  |  |  |  |  |  |  |
| CTLB1108-4 | Include | 2/6/1981 | F |  |  |  |  |  |  |  |
| CTLB1117-4 | Include | 2/18/1966 | F |  |  |  |  |  |  |  |
| CTLB1123-4 | Include | 7/20/1959 | F |  |  |  |  |  |  |  |
| CTLB1129-4 | Include | 5/26/1961 | F |  |  |  |  |  |  |  |
| CTLB1147-4 | Include | 4/1/1968 | M |  |  |  |  |  |  |  |
| CTLB1153-4 | Include | 7/15/1943 | F |  |  |  |  |  |  |  |
| CTLB1168-4 | Include | 1/25/1942 | F |  |  |  |  |  |  |  |
| CTLB1171-4 | Include | 11/29/1946 | M |  |  |  |  |  |  |  |
| CTLB1174-4 | Include | 12/8/1937 | F |  |  |  |  |  |  |  |
| CTLB1186-4 | Include | 3/8/1951 | M |  |  |  |  |  |  |  |
| CTLB0035-4 | Exclude | 17149 | F |  |  |  |  |  |  |  |
| CTLB0113-4 | Exclude | 19474 | M |  |  |  |  |  |  |  |
| CTLB0281-4 | Exclude | 18599 | M |  |  |  |  |  |  |  |
| CTLB0584-4 | Exclude | 16851 | F |  |  |  |  |  |  |  |
| CTLB0587-4 | Exclude | 19473 | M |  |  |  |  |  |  |  |
| CTLB0686-4 | Exclude | 14451 | M |  |  |  |  |  |  |  |
| CTLB0907-4 | Exclude | 23177 | F |  |  |  |  |  |  |  |
| CTLB0955-5 | Exclude | 17680 | M |  |  |  |  |  |  |  |
| CTLB0973-4 | Exclude | 22728 | M |  |  |  |  |  |  |  |
| CTLB1120-4 | Exclude | 15515 | F |  |  |  |  |  |  |  |
